# *SLG* controls grain size and leaf angle by modulating brassinosteroid homeostasis in rice

**DOI:** 10.1093/jxb/erw204

**Published:** 2016-06-01

**Authors:** Zhiming Feng, Chuanyin Wu, Chunming Wang, Jeehee Roh, Long Zhang, Jun Chen, Shengzhong Zhang, Huan Zhang, Chunyan Yang, Jinlong Hu, Xiaoman You, Xi Liu, Xiaoming Yang, Xiuping Guo, Xin Zhang, Fuqing Wu, William Terzaghi, Seong-Ki Kim, Ling Jiang, Jianmin Wan

**Affiliations:** ^1^State Key Laboratory for Crop Genetics and Germplasm Enhancement, Jiangsu Plant Gene Engineering Research Center, Nanjing Agricultural University, Nanjing 210095, China; ^2^National Key Facility for Crop Resources and Genetic Improvement, Institute of Crop Science, Chinese Academy of Agricultural Sciences, Beijing 100081, China; ^3^Department of Life Science, Chung-Ang University, Seoul 156-756, Korea; ^4^Department of Biology, Wilkes University, Wilkes-Barre, PA 18766, USA

**Keywords:** BR, grain size, homomer, leaf angle, rice, *SLG*.

## Abstract

Grain size and leaf angle are two important traits determining grain yield in rice. However, the mechanisms regulating the two traits remain largely unknown. Here, we characterized a rice gain-of-function mutant, *slender grain Dominant* (*slg-D*), which exhibited longer and narrower grains and larger leaf angles, similar to plants with elevated brassinosteroid (BR) levels or strengthened BR signaling. The increased cell length is responsible for the mutant phenotypes in *slg-D*. We demonstrated that the phenotype of *slg-D* is caused by enhanced expression of *SLG*, a BAHD acyltransferase-like protein gene. *SLG* is preferentially expressed in young panicles and lamina joints, implying its role in controlling cell growth in those two tissues. *slg-D* was restored to wild type by treatment with brassinazole, an inhibitor of BR biosynthesis. Overexpression of *SLG* in *d11-2* (deficient in BR synthesis) and *d61-1* (deficient in BR signaling) did not change the existing phenotypes. The *slg-D* plants had elevated BR contents and, accordingly, expression of BR-related genes was changed in a manner similar to BR treatment. Moreover, *SLG* RNAi plants displayed mild BR-deficient phenotypes including shorter grains, smaller leaf angles, and compact semi-dwarf plant types. The *in vitro* biochemical assays and transgenic approaches collectively demonstrated that SLG functions as homomers. Taken together, we conclude that *SLG* is an important regulator in BR homeostasis and that manipulation of *SLG* expression to an optimal level may provide a way to develop an ideal plant type.

## Highlight

The rice *SLG* gene, functioning as homomers, plays essential roles in regulating grain size and leaf angle via modulation of brassinosteroid homeostasis.

## Introduction

Rice (*Oryza sativa*) feeds more than half of the world’s population as one of the world’s most important cereal crops. Given the rapid increase in the world’s population and decrease in cultivated land area, improving rice production remains a great challenge for rice breeding programs. Grain size and leaf angle are two important traits determining rice grain yield and have always been a consideration in breeding programs (Sinclair and Sheehy, 1999; Ikeda *et al.*, 2013).

Grain size, determined by grain length, grain width, and grain thickness, not only contributes to grain yield, but also influences the appearance, processing, cooking, and eating quality of rice. For example, people in Japan, Korea, and Northern China favor medium length round grains, whereas people in the USA, Southeast Asian countries, and Southern China prefer long and slender grains (Unnevehr *et al.*, 1992). The organ size is largely determined by the cell number and cell size during organogenesis (Potter and Xu, 2001; Sugimoto-Shirasu and Roberts, 2003). In recent years, several genes and quantitative trait loci (QTLs) that affect grain size by influencing cell number have been identified in rice, including *GS3*, *GW2*, *GW5*, *GS5*, *GW8*, *qGL3*, *TGW6*, *GW6a*, and *BG1* (Song et al., 2007, 2015; Weng *et al.*, 2008; Mao *et al.*, 2010; Li *et al.*, 2011; Wang *et al.*, 2012; Zhang *et al.*, 2012; Ishimaru *et al.*, 2013; Liu *et al.*, 2015). Some other genes and QTLs that control grain size by influencing cell size have also been isolated in rice, including *PGL1*, *GL7*, and GS2/*GL2* (Heang and Sassa, 2012; Che *et al.*, 2015; Duan *et al.*, 2015; Hu *et al.*, 2015; Wang *et al.*, 2015). It has been documented that at least some of those genes participate in seed size control by regulating biosynthesis and signaling of plant hormones, including brassinosteroids (BRs), cytokinins, gibberellins, and auxin (Ashikari et al., 1999, 2005; Hong *et al.*, 2003; Tanabe *et al.*, 2005; Ishimaru *et al.*, 2013).

Leaf angle, the inclination between the leaf blade and vertical culm, is a key factor determining the plant architecture (Hoshikawa, 1989; Sinclair and Sheehy, 1999). A compact plant type with erect leaves is preferred since it increases photosynthetic efﬁciency and nitrogen storage for grain filling, and facilitates dense planting (Sinclair and Sheehy, 1999; Sakamoto *et al.*, 2006). A number of genes or QTLs have been reported to have a role in controlling leaf angle, including *Ta1*, *OsDWARF4*, *D2*, *OsBRI1*, *OsBU1*, *ILI1*, *LC2*, and *ILA1* (Li *et al.*, 1998, 1999; Sakamoto *et al.*, 2006; Tanaka *et al.*, 2009; Zhang *et al.*, 2009; Zhao *et al.*, 2010; Ning *et al.*, 2011). The leaf lamina joint that connects the leaf blade and sheath is considered the most important tissue governing the leaf angle. The degree of the leaf angle largely depends on cell division and expansion as well as cell wall composition at the joint (Nakamura *et al.*, 2009; Zhang *et al.*, 2009; Zhao *et al.*, 2010; Ning *et al.*, 2011). Nevertheless, it is well known that BR treatment stimulates leaf inclination in rice (Wada *et al.*, 1981).

BRs are a group of steroidal phytohormones that regulate diverse plant growth and developmental processes, including cell expansion and division, vasculature differentiation, root and leaf development, stem elongation, skotomorphogenesis, and grain ﬁlling (Clouse and Sasse, 1998; Fujioka and Yokota, 2003; Wu *et al.*, 2008). In recent decades, researchers have clarified many genes and the main pathway of BR biosynthesis utilizing genetic studies, chemical feeding, and enzymatic analysis. Most of the enzymes known to catalyze BR biosynthesis belong to the cytochrome P450 protein family (Choe, 2006). The BR biosynthesis pathway mainly consists of the early and late C-22 oxidation pathway, and the early and late C-6 oxidation pathway (Choe, 2006). Similarly, research on BR signaling has also developed rapidly, and most of the main participants in BR signaling have been determined in Arabidopsis (Belkhadir and Chory, 2006). BRs are perceived by the receptor kinase BRI1 to transmit signaling (Li and Chory, 1997). In rice, it has been reported that BR plays important roles in the regulation of grain size, leaf angle, and yield potential. For example, most loss-of-function mutants in BR biosynthesis or signaling pathways, such as *d2*, *d11*, and *d61*, display short grains, erect leaves, and dwarf phenotypes (Yamamuro *et al.*, 2000; Hong *et al.*, 2003; Tanabe *et al.*, 2005), while some other mutants or transgenic plants with enhanced BR signaling or increased BR levels, such as *GSK2* knockdown lines, the *DLT* overexpresser, and the *D11* activation mutant *m107*, show longer grains and larger leaf angles (Tanabe *et al.*, 2005; Wan *et al.*, 2009; Tong *et al.*, 2012). More importantly, modulating the expression level of BR-related genes such as *OsDWARF4* and *OsBRI1* has been proven to improve rice grain yield at higher planting densities (Morinaka *et al.*, 2006; Sakamoto *et al.*, 2006).

BR homeostasis is vital for normal growth and development of plants. BRs are synthesized in most plant tissues, and their level is the highest in young developing organs but low in mature organs (Shimada *et al.*, 2003). Unlike the other plant hormones such as auxin that can be transported from the site of synthesis to a distant target site (Berleth and Sachs, 2001), BRs do not undergo long-distance transport and have the same site of synthesis and action (Symons and Reid, 2004). Therefore, there exist mechanisms that cells or tissues use to modulate levels of endogenous BRs precisely to keep cell expansion in balance and ensure normal plant growth and development (Symons and Reid, 2004). Negative feedback regulation is a common mechanism that also regulates BR homeostasis. It is reported that expression of many BR biosynthesis and signaling genes is inhibited by BR treatment, such as *D2*, *D11*, *OsDWARF4*, *BRD1*, *OsBRI1*, and *DLT* in rice (Yamamuro *et al.*, 2000; Hong et al., 2002, 2003; Tanabe *et al.*, 2005; Sakamoto *et al.*, 2006; Tong *et al.*, 2009). However, BR homeostasis is still poorly understood.

In this study, we characterized a rice semi-dominant mutant, *slender grain Dominant* (*slg-D*), with slender grains and enlarged leaf angles, which are caused by enhanced expression of *SLG*, a BAHD acyltransferase-like protein gene. We provide genetic evidence that the BR contents are associated with the expression level of *SLG*. In addition, the plants expressing an RNAi or a truncated version of *SLG* showed a semi-dwarf architecture with smaller leaf angles, which may be useful for rice yield improvement.

## Materials and methods

### Plant materials

The *slg-D* mutant (3A-10513) was isolated from a collection of activation-tagging T-DNA insertion rice lines (Jeon *et al.*, 2000; Jeong *et al.*, 2006), and kindly provided by Professor Gynheung An. The wild type (WT) of *slg-D* was Dongjin, a *japonica* cultivar. *d61-1*, *d11-2*, and *m107* were kindly provided by Professor Chencai Chu (Tong *et al.*, 2012). The WT of *d61-1* and *d11-2* was a *japonica* cultivar, Zhonghua11, and the WT of *m107* was a *japonica* cultivar, Nipponbare. Rice plants were cultivated in an experimental ﬁeld under natural long-day conditions in Nanjing, China.

### Scanning electron microsocpy (SEM) and light microscopy

For SEM, lemmas were harvested from florets after flowering and ﬁxed in 2.5% (v/v) glutaraldehyde. Fixed samples were soaked in 2% (w/v) OsO4 for 2h, dehydrated in a graded ethanol series, infiltrated and embedded in butyl methyl methacrylate, treated with critical point drying, and then sputter coated with platinum. The outer and inner epidermal cells of lemmas were observed using a HITACHI S-3400N scanning electron microscope. For light microscopy, lamina joints of the second leaves were harvested 10 d after ﬂowering and fixed with FAA solution, followed by a graded series of dehydration and infiltration steps. Fixed tissues were embedded in paraplast. After sectioning, 10 μm thick sections were dewaxed with xylene, rehydrated, stained with 1% toluidine blue, and observed with a Leica DM5000B microscope. Cell lengths and widths of each organ were measured with IMAGEJ software.

### Isolation, cloning, and RNAi suppression of the *SLG* gene

To identify the T-DNA insertion locus in *slg-D*, we searched the ﬂanking sequence database (Jeong *et al.*, 2006; http://orygenesdb.cirad.fr/). The T-DNA loci were conﬁrmed by PCR genotyping, using the primers P1, P2, and P3 (see Supplementary Table S1 at *JXB* online). To recapitulate the phenotype of *slg-D*, full-length cDNAs of *Loc_Os08g44830* and *Loc_Os08g44840* were ampliﬁed by PCR and cloned into the binary vector pCUbi1390 under the control of the maize *Ubi* promoter to create p1390-Ubi-*830*, and p1390-Ubi-*840* constructs, respectively. These constructs were then transformed into the rice variety Dongjin according to a published *Agrobacterium*-mediated method (Hiei *et al.*, 1994).

To obtain *SLG* RNAi plants, the construct pCUbi1390-ΔFAD2 (an FAD2 intron and ubiquitin promoter inserted into pCUbi1390) was used as an RNAi vector (Wu *et al.*, 2007). Both sense and antisense versions of a speciﬁc 305bp fragment from the cDNA of *SLG* were ampliﬁed with primer pairs SLG-RNAiL and SLG-RNAiR (Supplementary Table S1), and cloned into pCUbi1390-ΔFAD2 to create the pUbi-dsRNAiSLG construct, which was then transformed into the rice variety Dongjin by the *Agrobacterium*-mediated method described above.

### RNA extraction and quantitative RT-PCR

Total RNA from roots, leaves, leaf sheaths, lamina joints, shoot apices, culms, and different stages of panicles were isolated using the RNAprep Pure Plant Kit (TIANGEN, Beijing, China). First-strand cDNA was reverse transcribed from 1 μg of total RNA using the PrimeScript 1st Strand cDNA Synthesis Kit (TaKaRa). Quantitative RT-PCR was performed using a SYBR Premix Ex TaqTM kit (TaKaRa) on an ABI prism 7500 Real-Time PCR System according to the manufacturer’s instructions, and the *ACTIN1* gene was used as an internal control. The primers for quantitative RT-PCR analysis are listed in Supplementary Table S1.

### GUS staining

To analyze the expression pattern of *SLG*, an ~2.5kb promoter fragment was cloned into the pCAMBIA1381Z vector to create the *PRO*_*SLG*_ *:GUS* (β-glucuronidase) reporter gene construct, which was then transformed into the rice variety Dongjin by the *Agrobacterium*-mediated method. GUS staining was performed on *PRO*_*SLG*_ *:GUS* T_1_ generation transgenic plants according to a method described previously (Jefferson *et al.*, 1987). Images were taken using a Nikon CD5Ri1P camera. Primers used to clone the promoter fragment are listed in Supplementary Table S1.

### *In situ* hybridization

RNA *in situ* hybridization was performed as described previously (Bradley *et al.*, 1993). A 305bp gene-specific region of *SLG* amplified with primers SLG-PF and SLG-PR (see Supplementary Table S1) was cloned into the pGEM-T Easy vector (Promega). The linearized templates were amplified from the pGEM-T plasmid containing the gene-specific region of *SLG* using primers Yt7 and Ysp6. Digoxigenin-labeled RNA probes were transcribed in vitro using T7 and SP6 RNA polymerases, respectively, using a DIG Northern Starter Kit (Cat. No. 2039672, Roche) following the manufacturer’s instructions. Images were taken using a Leica DM5000B microscope.

### Subcellular localization of SLG

To determine the subcellular localization of SLG, green fluorescent protein (GFP) was fused to the C-terminus of SLG under the control of the 35S promoter in the PAN580 vector. In addition, the nuclear marker D53–mCherry was constructed. The SLG–GFP fusion construct was transiently co-transferred into rice protoplasts with the D53–mCherry constructs according to the method described previously (Chen *et al.*, 2006). Next, GFP was fused to the C-terminus of SLG under the control of the *Cauliflower mosiac virus* (CaMV) 35S promoter in the pCAMBIA1305.1 vector. The pCAMBIA1305-SLG-GFP construct was transformed into the rice variety Dongjin by the *Agrobacterium*-mediated method. GFP ﬂuorescence was examined in the young roots of 2-week-old T_1_ transgenic plants. Fluorescence images were observed using a Zeiss LSM510 confocal laser microscope. Primers used to make these constructs are listed in Supplementary Table S1.

### BR and BRZ treatment

The lamina joint bending assay using excised leaf segments was performed as described by Wada *et al.* (1981). Seeds were germinated for 2 d and then grown in the dark for 8 d at 30 °C. Segments of 2cm comprising the second leaf blade, lamina joint, and leaf sheath were ﬂoated on distilled water for 24h and then incubated in 2.5mM maleic acid potassium solution containing various concentrations of brassinolide (BL; Sigma, http://www.sigmaaldrich.com/) for 48h in the dark. Lamina joint angles were measured using IMAGEJ software. The coleoptile and root elongation tests were performed using a previously described method (Yamamuro *et al.*, 2000). Seeds were germinated on agar plates containing various concentrations of BL, and then the coleoptile and root lengths were measured 1 d after germination.

To measure the effect of brassinazole (BRZ; TCI) treatment on lamina joint bending, the leaf tips of 8-day-old seedlings of *slg-D* and the WT were spotted with 1 μl of DMSO containing 0 or 10 μM BRZ daily for 3 d, followed by 7 d growth in a controlled growth chamber under long-day conditions (16h light at 28 °C/8h darkness at 24 °C). The angles of the third lamina joints were measured using IMAGEJ software.

### Measuring endogenous BRs

BR contents were analyzed using gas chromatography–mass spectrometry (GC-MS) as described previously (Kim *et al.*, 2005). Four-week-old shoots of the WT (88.50g FW) and *slg-D* (65.43g FW) were harvested, lyophilized, and extracted three times with 500ml of 80% methanol. Deuterium-labeled 6-deoxocastasterone (6-deoxoCS), typhasterol (TY), castasterone (CS), and BL were added as internal standards for quantitative analysis of the extracts (200ng each).

### Yeast two-hybrid assay

The full-length cDNA of *SLG* was cloned into pGBKT7 (Clontech, http://www.clontech.com). Full-length *SLG* as well as its N- and C-terminal truncated deletions were then subcloned into pGADT7 (Clontech) and all vectors were transformed into yeast strain AH109. A yeast two-hybrid library was constructed from the mRNA of young rice panicles 0.1–5cm long. Yeast transformation and screening procedures were performed according to the manufacturer’s instructions (Clontech). Primers used to make these constructs are listed in Supplementary Table S1.

### Bimolecular fluorescence complementation (BiFC) assay

The full-length *SLG* cDNA was cloned into the vector pSPYCE(M), and the *SLG* cDNA and its truncated deletions were then subcloned into the vector pSPYNE173. The plasmids were transiently expressed in *Nicotiana benthamiana* leaves as described previously (Waadt and Kudla, 2008). Yellow fluorescent protein (YFP) ﬂuorescent signals were observed under a Zeiss LSM510 confocal laser microscope between 48h and 72h post-transfection. Primers used to make these constructs are listed in Supplementary Table S1.

### Pull-down assay

The *SLG* cDNA was cloned into the vectors pMAL-c2x and pGEX4T-2 to generate fusions with maltose-binding protein (MBP) and glutathione *S*-transferase (GST), respectively. Expression of MBP–SLG, GST–SLG, and GST in BL21 Rosetta cells was induced with 0.5mM isopropyl-β-d-thiogalactoside at 16 °C for 20h. The total protein concentration was quantiﬁed using the Bio-Rad protein assay reagent. The pull-down assay was performed as reported previously (Miernyk and Thelen, 2008). The proteins were separated on a 10% SDS–PAGE gel and immunoblotted with anti-GST or anti-MBP antibodies (Abmart, http://www.ab-mart.com). The primers used to make these constructs are listed in Supplementary Table S1.

## Results

### Phenotypic characterization of the semi-dominant mutant *slg-D*

**Fig. 1.**
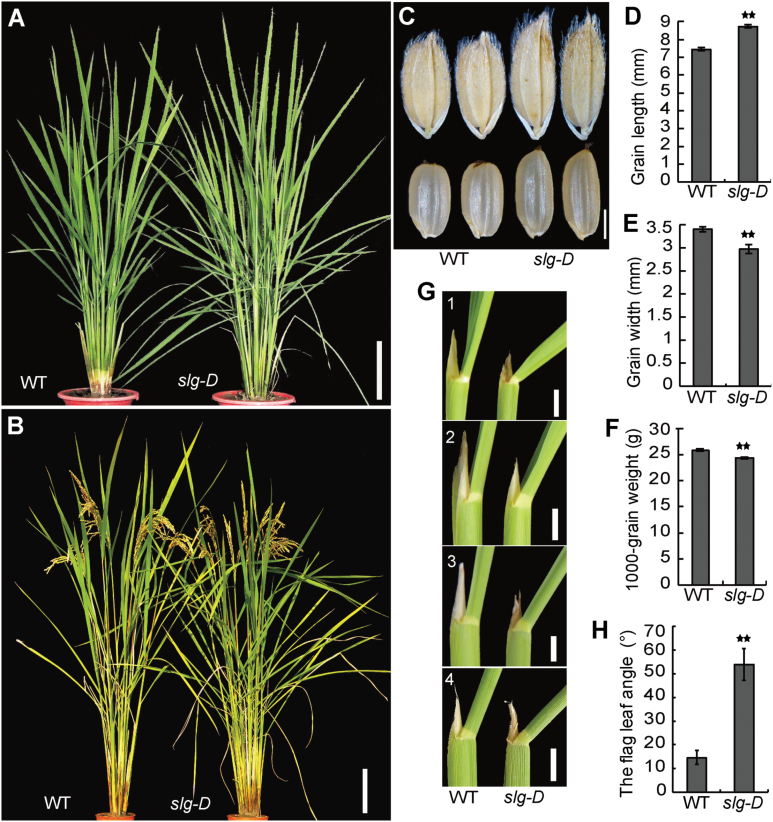
Phenotype of the *slg-D* mutant. (A, B) Gross morphologies of the WT and *slg-D* at the vegetative (A) and mature (B) stages. (C) Slender grains of *slg*-D compared with the WT. (D–F) Measurements of traits showing longer but narrower grains (D and E), and reduced seed weight (F) in *slg*-D. (G) Comparison of the lamina joint angle of the ﬂag (1), second (2), third (3), and fourth (4) leaves between *slg*-D and the WT (counted from the ﬂag leaf downwards). (H) Quantitation of the flag leaf lamina joint angles. Values are given as means ±SD (*n*=10 in D–F, H). **P<0.01 compared with the WT by Student’s *t*-test. Scale bars=10cm (A, B), 2mm (C), or 2cm (G).

To identify new components involved in regulating rice grain size, we screened a collection of activation-tagging T-DNA insertion rice lines (Jeon *et al.*, 2000; Jeong *et al.*, 2006). As a result, we isolated a mutant (3A-10513) with a slender-grain phenotype, and named it *slender grain Dominant* (*slg-D*). *slg-D* showed less compact plant architecture than the WT at both the vegetative and mature stages (Fig. 1A, B). In *slg-D*, the grain length was significantly increased while the grain width decreased, and the 1000-grain weight was slightly decreased (Fig. 1C–F). The lamina joint bending angles of *slg-D* were larger than those of the WT, especially for the ﬂag leaves (Fig. 1G, H). Together, these results indicate that *slg-D* displays slender grain and an enlarged leaf angle.

The F_1_ plants from the cross *slg-D*×WT exhibited an intermediate phenotype in grain shape and leaf angle, indicating a semi-dominant nature of the mutation (Supplementary Fig. S1A–F). Genetic analyses of an F_2_ population derived from the same cross showed a segregation ratio of 1:2:1 (64 normal:120 intermediate:56 mutant; χ^2^=0.09, *P>*0.05), suggesting that *slg-D* is a single locus mutation (Supplementary Fig. S1G). This observation provides a hint that the semi-dominant nature of *slg-D* might be associated with an insertion of the activation-tagging T-DNA.

### Cell length change in *slg-D* determines the mutant phenotypes

**Fig. 2.**
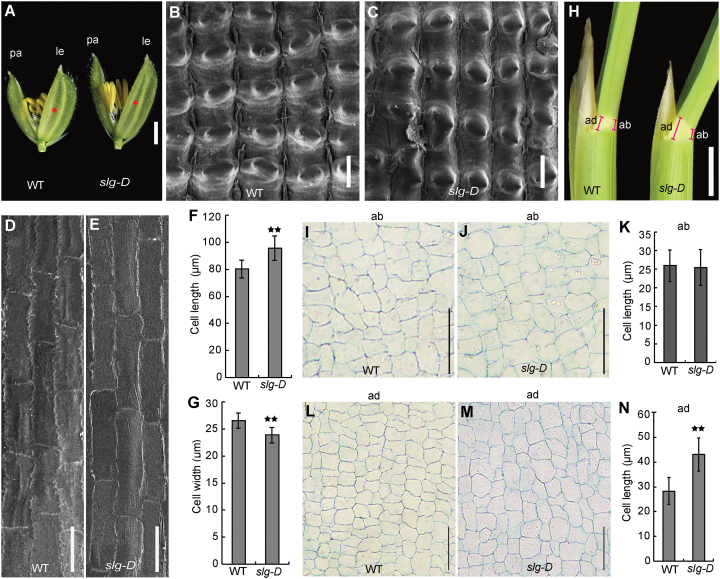
The changes in cell length are responsible for the phenotype in *slg-D*. (A) Spikelets just before heading. The red asterisk indicates the site used for detailed observation in (B–E). pa, palea; le, lemma. (B, C) SEM images of outer epidermal cells of lemmas. (D, E) SEM images of inner epidermal cells of lemmas. (F, G) Average length (F) and average width (G) of inner epidermal cells of lemmas. (H) Comparison of second leaf lamina joints (counted from the ﬂag leaf downwards) between the WT and *slg-D*. Red lines indicate cut sites. ad, adaxial; ab, abaxial. (I, J) Longitudinal sections of the abaxial sides of the second leaf lamina joint shown in (H). (K) Average lengths of cells shown in (I) and (J). (L, M) Longitudinal sections of the adaxial sides of the second leaf lamina joints shown in (H). (N) Average lengths of cells shown in (L) and (M). Values are given as means ±SD (*n*=3 in F, G, K, N). **P<0.01 compared with the WT by Student’s *t*-test. Scale bars=2mm (A), 50 μm (B–E), 2cm (H), or 100 μm (I, J, L, M).

To investigate the mutant phenotypes in *slg-D* at a cell level, we performed SEM and light microscopy on *slg-D* plants along with the WT. The observations on the outer and inner epidermal cells of lemmas, which determine the shape and size of grains, showed that those cells were stretched longitudinally in *slg-D*, such that the slender-grain phenotype was developed (Fig. 2A–G). The histological analysis on the second leaf lamina joints indicated that there was no signiﬁcant alteration in cell size in the abaxial sides between *slg-D* and the WT (Fig. 2H–K). In contrast, the cell length of the adaxial sides was increased in *slg-D* (Fig. 2H, L–N). Therefore, it is the asymmetric cell expansion at the opposite sides of the lamina joint that causes a larger leaf bending in *slg-D*. Overall, these results indicate that the changes in cell length are responsible for the phenotype in *slg-D*.

### Enhanced expression of *Loc_Os08g44840* in *slg-D* leads to the mutant phenotypes

**Fig. 3.**
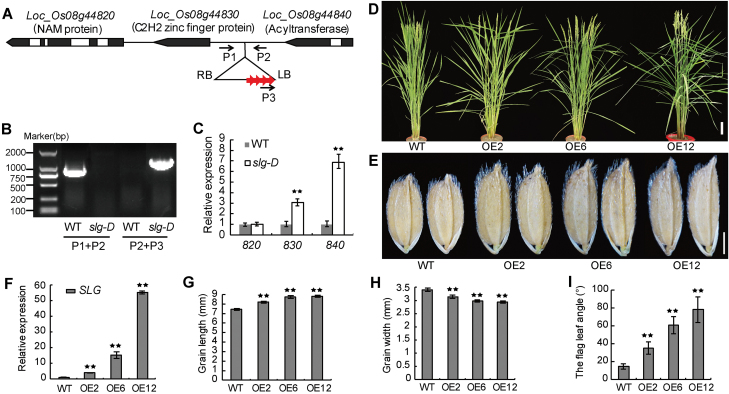
Enhanced expression of *Loc_Os08g44840* in *slg-D* leads to the mutant phenotypes. (A) T-DNA insertion site in *slg-D*. The four copies of the 35S enhancers in the T-DNA are indicated with red arrows, and white boxes indicate introns. P1, P2, and P3 indicate primers used for verification of the insertion site. RB and LB indicate the T-DNA right and left borders, respectively. (B) PCR verification of the insertion site. (C) Quantitative RT-PCR analysis of expression of genes adjacent to the T-DNA insertion site. (D) Gross appearance of *pUbi::Loc_Os08g44840* transgenic plants recapitulating the *slg-D* phenotype in the WT background. OE2, OE6, and OE12 are three independent lines. (E) Grains of the plants shown in (D). (F) *SLG* expression levels in the plants shown in (D). (G, H) Average lengths (G) and widths (H) of grains shown in (E). (I) Quantitation of the flag leaf angles shown in (D). Values are given as means ±SD (*n*=3 in C, F; *n*=10 in G–I). **P<0.01 compared with the WT by Student’s *t*-test. Scale bars=10cm (D) or 2mm (E).

To isolate the gene for *slg-D*, we searched the T-DNA insertion database and obtained a genomic ﬂanking sequence (Jeong *et al.*, 2006; http://orygenesdb.cirad.fr/). Based on this information, we designed three PCR primers (P1, P2, and P3) and confirmed the site of the T-DNA insertion in *slg*-D (Fig. 3A, B). To understand whether the mutation in *slg-D* is related to an insertion of the activation-tagging T-DNA, we PCR-genotyped the mutant F_2_ plants derived from the cross *slg-D*×WT and found that the mutant phenotypes were always associated with the presence of the T-DNA (Supplementary Fig. S2). A BLAST search (http://www.ncbi.nlm.nih.gov/) showed that the T-DNA was inserted in an intergenic region, with *Loc_Os08g44830* 2000bp upstream and *Loc_Os08g44840* 4500bp downstream (Fig. 3A). Another nearby gene is *Loc_Os08g44820*, upstream of *Loc_Os08g44830* (Fig. 3A). Next we examined if the four enhancer repeats in the T-DNA had an influence on the expression level of the three nearby genes, and found that two of them (*Loc_Os08g44830* and *Loc_Os08g44840*) had elevated expression (Fig. 3C). This result suggests that the changed expression level of the two genes might be responsible for the mutant phenotypes in *slg-D*.

We assumed that overexpression of *Loc_Os08g44830*, *Loc_Os08g44840*, or both in the WT might recapitulate the phenotypes observed in *slg-D*. To test this assumption, *Loc_Os08g44840* and *Loc_Os08g44830*, under control of the maize *Ubi* promoter, were individually overexpressed in WT plants. Interestingly, only plants overexpressing *Loc_Os08g44840*, not *Loc_Os08g44830*, showed varying degrees of enlarged leaf angle and slender grain, thus phenocopying *slg-D* (Fig. 3D–I; Supplementary Fig. S3). The phenotypic variation in the transgenic plants was well correlated with the expression level of *Loc_Os08g44840* (Fig. 3D–I). We concluded that it is the enhanced expression of *Loc_Os08g44840* that causes the phenotypic changes in *slg-D*. We designated *Loc_Os08g44840* as a *SLENDER GRAIN* (*SLG*) gene.

### *SLG* encodes a putative BAHD acyltransferase-like protein

*SLG* encodes a protein of 445 amino acids. SLG belongs to the putative BAHD family of acyltransferases, which catalyze formation of a diverse group of plant metabolites using CoA thioesters as substrates (D’Auria, 2006). A phylogenetic analysis revealed that SLG-like proteins largely fall into two groups: dicot and monocot, and SLG is a member of the monocot group (Supplementary Fig. S4). However, to date, none of the genes in this group has been functionally characterized.

The BAHD enzymes typically contain two highly conserved domains: the HXXXDG motif (or HXXXDA) located near the center, and the DFGWG motif located near the C-terminus (D’Auria, 2006). The two conserved motifs in SLG are HAVLDG (167–172) and DFGFG (386–390) with a substitution of tryptophan by phenyalanine (Supplementary Fig. S5).

### Expression pattern and subcellular localization of *SLG*

**Fig. 4.**
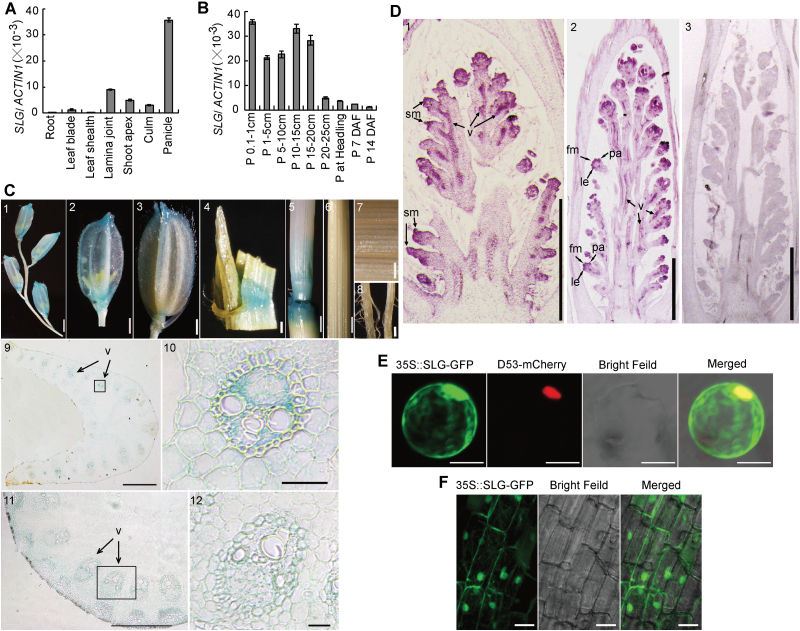
Spatiotemporal expression and subcellular localization of SLG. (A) Quantitative RT-PCR analysis of *SLG* expression in various rice tissues. Roots, leaf blades and sheaths, lamina joints, and culms were harvested from plants at the early booting stage. Shoot apices were collected from 4-week-old seedlings. Panicles were collected when they had reached 1cm length. (B) *SLG* expression in panicle tissues at various stages. P, panicle; DAF, day after flowering. Data (A, B) are ratios of *SLG* to *ACTIN1* signals and are presented as means ±SD (*n*=3). (C) GUS staining of various *PRO*_*SLG*_ *:GUS* transgenic line tissues. (1) Young spikelet. (2) A young ﬂoret in (1). (3) A mature ﬂoret after flowering. (4) Lamina joint. (5) The bottom portion of the young second internode and stem node. (6) The top portion of the young second internode. (7) Mature leaf blade. (8) Mature root. (9) Cross-section of the lamina joint shown in (4). (10) Magniﬁed image of the region enclosed by the square in (9). (11) Cross-section of the lower part of the tissue shown in (5). (12) Magniﬁed image of the region enclosed by the square in (11). Scale bars=1mm (1–4, 7–9, 11), 1cm (5, 6), or 100 μm (10, 12). (D) *In situ* localization of *SLG* mRNA. (1) An early stage of panicle development. (2) A late stage of panicle development. (3) *SLG in situ* hybridization negative control using a sense probe. Scale bars=1mm (1–3). v, vasculature; sm, spikelet meristem; le, lemma; pa, palea; fm, ﬂoral meristem. (E) Transient expression of SLG–GFP and D53–mCherry fusion proteins in rice leaf sheath protoplasts. Scale bar=5 μm. (F) Fluorescent signals in transgenic root cells expressing the SLG–GFP fusion protein. Scale bar=30 μm.

Our quantitative RT-PCR analysis showed that expression of *SLG* is strong in young panicles, relatively high in lamina joints, low in shoot apices, culms and leaves, and very little in roots and leaf sheaths (Fig. 4A). Further, *SLG* has the highest expression level during early panicle development, but drops dramatically as the spikelets reach their final size (Fig. 4B). To investigate further the expression pattern of *SLG*, a genomic sequence ~2.5kb upstream of the translation start site was cloned and introduced into the pCAMBIA1381Z vector, resulting in the *PRO*_*SLG*_ *:GUS* reporter construct. Analysis of GUS activity in transgenic lines showed that strong GUS staining was observed in young panicles, lamina joints, and young stem nodes, with faint staining in leaf veins, but not visible in roots and leaf sheaths (Fig. 4C, parts 1–8). Cross-sectioning of the GUS-stained leaf lamina joint and the bottom portion of the stained internode further showed that the GUS signals were mainly restricted to vasculature regions (Fig. 4C, parts 9–12). We also performed RNA *in situ* hybridization to localize *SLG* expression during early panicle development more precisely. Strong *SLG* expression was detected in spikelet meristem primordia, ﬂoral meristem primordia, lemma and palea primordia, and vasculature regions (Fig. 4D). The predominant expression of *SLG* in young panicles and lamina joints implies its role in controlling grain shape and leaf angle.

To determine the subcellular localization of SLG, we fused the green ﬂuorescent protein (GFP) to the C-terminus of SLG. Transient expression of this fusion protein in rice protoplasts revealed that the GFP signals were found in both the cytoplasm and the nucleus (Fig. 4E). Transgenic rice plants harboring the same fusion construct also showed the cytoplasmic and nuclear localization pattern of SLG (Fig. 4F).

### *SLG* positively regulates endogenous BR levels

**Fig. 5.**
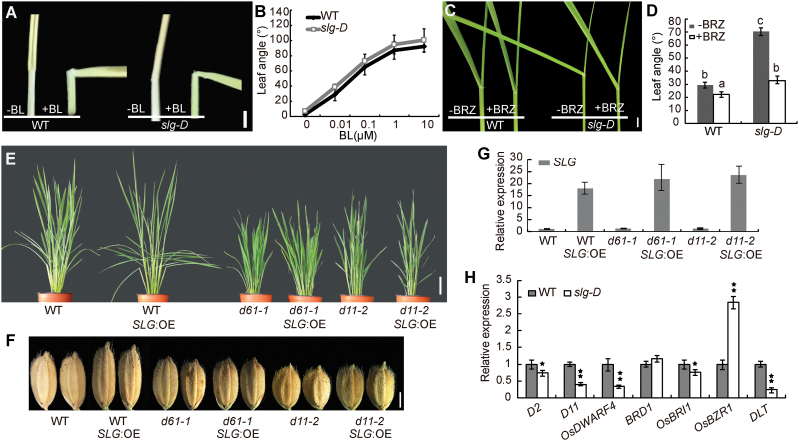
*SLG* is involved in regulating endogenous BR levels but not BR signaling. (A) Response of the second leaf lamina joint to 10 μM BL treatment by the excised leaf segment method. (B) Dose response of the bending angle to various concentrations of BL. (C) Response of the third leaf lamina joint to 10 μM BRZ. (D) Measurement of the lamina joint angles after the 10 μM BRZ treatment shown in (C). (E, F) Gross morphologies (E) and grains (F) of WT, WT *SLG*:OE, *d61-1*, *d61-1 SLG*:OE, *d11-2*, and *d11-2 SLG*:OE. (G) Expression levels of *SLG* in the different lines shown in (E). (H) Quantitative RT-PCR analysis of BR-related genes in young panicles of *slg-D* and the WT. Values are given as means ±SD (*n*=10 in B, D; *n*=3 in G, H). Different letters (D) indicate *P*<0.01 (LSD multiple range tests). *P<0.05; **P<0.01 (H) compared with the WT by Student’s *t*-test. Scale bars=2cm (A, C), 10cm (E), or 2mm (F).

The *slg-D* phenotype resembles that of an activation mutant or transgenic plants with elevated BR accumulation (Wu *et al.*, 2008; Wan *et al.*, 2009), and that of transgenic plants with enhanced BR signaling (Tanaka *et al.*, 2009; Tong *et al.*, 2012), leading us to hypothesize that *SLG* may be involved in regulating the BR pathway. To determine whether *slg-D* responds differently to BR treatment, we first performed lamina joint bending assays using excised leaf segments (Wada *et al.*, 1981). We measured the effects of a range of 24-epibrassinolide (BL; a type of active BR) concentrations on the angle of the lamina joints, and found that lamina joint bending was increased in a dose-dependent manner and the sensitivity to BL treatment was similar in *slg-D* and the WT (Fig. 5A, B). Next we performed another BR response assay involving coleoptile and root elongation using a previously described method (Yamamuro *et al.*, 2000). Comparison of coleoptile and root lengths also showed that *slg-D* had a similar response to BL as the WT (see Supplementary Fig. S6). These results indicate that BR signaling is not altered in *slg-D*.

To investigate whether *SLG* functions in regulating endogenous BR levels, we first tested the effect of brassinazole (BRZ; a specific BR biosynthesis inhibitor; Asami *et al.*, 2000) on *slg-D* in a lamina joint bending experiment. The leaf tips of 8-day-old seedlings of *slg-D* and the WT were spotted with 10 μM BRZ daily for 3 d, followed by 7 d growth in a chamber. We observed that the leaf angle of *slg-D* was restored to the WT level by BRZ treatment, whereas the WT seedlings had a milder response to the same treatment (Fig. 5C, D), indicating that *slg-D* is more sensitive to BRZ. A similar result was also seen when the *D11* activation line *m107*, a BR overproduction mutant, was treated with BRZ (see Supplementary Fig. S7). Next we introduced the *SLG*-overexpressing construct into *d61-1* (a loss-of-function mutant of the BR receptor gene *OsBRI1*; Yamamuro *et al.*, 2000) and *d11-2* (a mutant deficient in BR biosynthesis; Tanabe *et al.*, 2005), and found that the *d61-1 SLG:OE* and *d11-2 SLG:OE* plants still retained the dwarfism, smaller and round grains, and erect leaves (Fig. 5E–G). Those results suggest a role for *SLG* in regulating BR levels. Consistent with this, our chemical analysis indeed showed a higher content of 6-deoxocastasterone (6-deoxoCS), typhasterol (TY), and castasterone (CS) in *slg-D* than in the WT (Supplementary Fig. S8).

It is known that excessive BRs down-regulate the BR-related genes *D2*, *D11*, *OsDWARF4*, *BRD1*, *OsBRI1*, and *DLT*, but up-regulate *OsBZR1* as a feedback mechanism (Yamamuro *et al.*, 2000; Hong et al., 2002, 2003; Wang *et al.*, 2002; He *et al.*, 2005; Tanabe *et al.*, 2005; Sakamoto *et al.*, 2006; Tong *et al.*, 2009). We analyzed the expression level of those genes in *slg-D* and found that all except *BRD1* had the expected transcription level change as a response to the elevated BR levels (Fig. 5H). As an alternative control, we also measured expression of those BR genes in the mutant *m107*, where *D11* was dramatically enhanced and BR levels increased, and found similar expression changes (see Supplementary Fig. S9). Those results further confirm higher BR contents in *slg-D*. However, *SLG* itself did not respond to the exogenous BL treatment (Supplementary Fig. S10).

Taken together, these results suggested that *SLG* positively regulates endogenous BR levels and is a new regulator of BR homeostasis in rice.

### Suppression of *SLG* leads to BR-deficient phenotypes

**Fig. 6.**
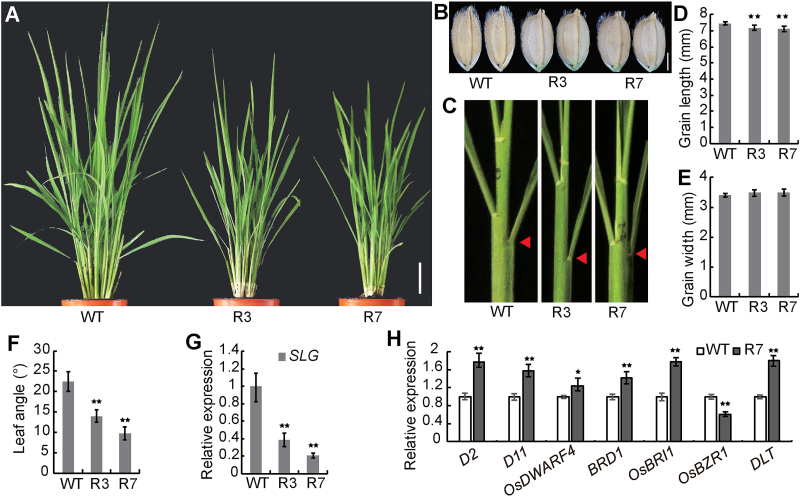
Phenotypes of *SLG* RNAi transgenic plants. (A–C) Gross morphologies (A), grains (B), and leaf angles (C) of *SLG* RNAi transgenic plants in the WT background. R3 and R7 are two independent lines. (D–F) Quantitative comparisons of grain lengths (D), grain widths (E), and leaf angles (F) of the lines shown in (A). Leaf angles were measured at the positions indicated by the arrowheads in (C). (G) *SLG* expression in the lines shown in (A). (H) Quantitative RT-PCR analysis of BR-related genes in R7 and the WT. Values are given as means ±SD (*n*=10 in D–F; *n*=3 in G, H). *P<0.05; **P<0.01 compared with the WT by Student’s *t*-test. Scale bars=10cm (A) or 2mm (B).

To explore further the function of *SLG*, a *SLG* RNAi vector was constructed and introduced into WT plants. The *SLG* RNAi plants displayed a more compact architecture, reduced plant height, smaller leaf angle, and shorter and rounder grain (Fig. 6A–G). These phenotypes are similar to those of BR-deficient mutants, such as *d61* and *d11* (Hong *et al.*, 2003; Tanabe *et al.*, 2005). In addition, we investigated expression changes of the genes involved in BR synthesis or signaling in R7, a typical *SLG* RNAi line with greatly reduced leaf angle and *SLG* expression (Fig. 6F, G). Six of the genes detected, *D2*, *D11*, *OsDWARF4*, *BRD1*, *OsBRI1*, and *DLT* were up-regulated, but *OsBZR1* was down-regulated in R7 compared with the WT (Fig. 6H). The feedback regulation of those BR-related genes caused by knock-down of *SLG* further supports involvement of *SLG* in regulating the BR level. These results further highlight a role for *SLG* in regulating BR homeostasis. This observation also suggests that an optimized expression level of *SLG* may help create a compact and semi-dwarf ideal plant type.

### SLG functions as homomers

**Fig. 7.**
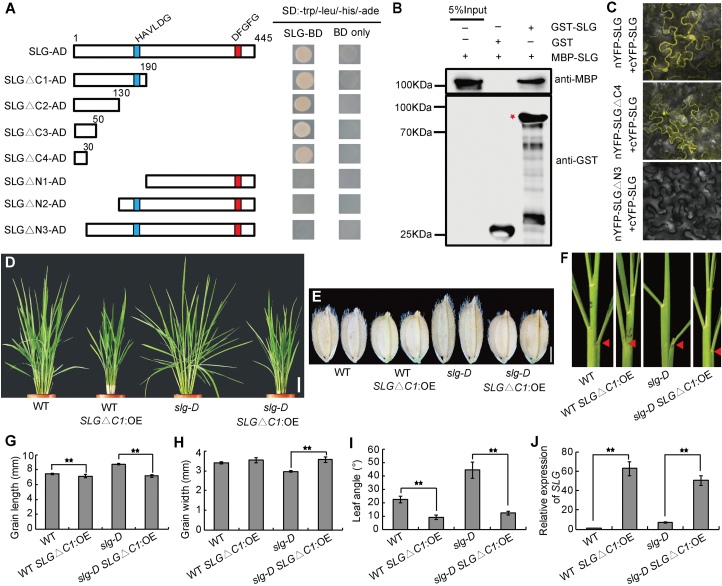
SLG functions as homomers. (A) Yeast two-hybrid assays. Schematic representations of the truncated SLG proteins used for the yeast two-hybrid assays. Cyan and red rectangles represent the two conserved motifs. ∆N and ∆C represent several N- and C-terminally truncated SLG deletions. (B) Pull-down assay showing direct interaction between MBP–SLG and GST–SLG in vitro. (C) BiFC assay showing that cYFP–SLG and nYFP–SLG interacted to form a functional YFP in tobacco leaf cells. (D–F) Gross morphologies (D), grains (E), and leaf angles (F) of WT, WT *SLG∆C1*:OE, *slg-D*, and *slg-D SLG∆C1*:OE plants. (G–I) Quantitative comparisons of grain lengths (G), grain widths (H), and leaf angles (I) of the lines shown in (D). Leaf angles were measured at the positions indicated by the arrowheads in (F). (J) Expression levels of *SLG* in the lines shown in (D). Values are given as means ±SD (*n*=10 in G–I; *n*=3 in J). *P<0.05; **P<0.01 by Student’s *t*-test. Scale bars=10cm (D) or 2mm (E).

It has been reported that enzyme proteins often function as homomers or heteromers (Ali and Imperiali, 2005). To investigate the functional forms of SLG, the full-length SLG protein was used as a bait to screen a yeast two-hybrid library prepared from young rice panicles. We identiﬁed four positive clones that contain different *SLG* cDNA fragments from ~1 million yeast transformants. To conﬁrm the self-interaction of SLG, different truncated SLG proteins were used for interaction analysis. As shown in Fig. 7A, a 30 amino acid region in the N-terminus of SLG (SLG∆C4), rather than the two conserved motifs, was required for the self-interaction of SLG. An *in vitro* GST pull-down assay also conﬁrmed the self-interaction (Fig. 7B). In addition, BiFC analysis also showed that SLG physically interacted with itself and this interaction required the N-terminal 30 amino acid region (Fig. 7C).

To study the importance of SLG self-interaction, two truncated *SLG* CDS, *SLG∆C1* with only the 190 N-terminal amino acids, and *SLG∆N3* without the 30 N-terminal amino acids, were individually overexpressed in *slg-D* and the WT. We found that overexpression of *SLG∆C1* but not *SLG∆N3* in both the WT and *slg-D* resulted in shorter and rounder grains, smaller leaf angles, and dwarf phenotypes, similar to those of *SLG* RNAi plants (Fig. 7; Supplementary Fig. S11). The truncated SLG∆C1 protein might interfere with formation of functional homomers between the intact SLG proteins, thus leading to a dominant negative mutant phenotype. When the interaction region was removed in SLG∆N3, however, the truncated protein did not exert any effect on SLG, thus providing genetic evidence that SLG indeed functions as homomers *in vivo*.

## Discussion

In this study, we have provided evidence that *SLG* is involved in BR homeostasis by positively regulating endogenous BR levels to control grain size and leaf angle in rice. First, the activation-tagging mutant *slg-D* and transgenic plants overexpressing *SLG* displayed longer and narrower grains and larger leaf angles that are similar to the mutants or transgenic plants with enhanced BR signaling or increased BR levels. Secondly, *slg-D* and the WT had similar sensitivity to BL treatment in lamina joint bending, coleoptile elongation, and root elongation assays. Thirdly, the BRZ treatment restored *slg-D* to the WT. Fourthly, overexpression of *SLG* in the BR-related mutants, *d61-1* and *d11-2*, did not lead to slender grains and enlarged leaf angles. Fifthly, the major BRs were increased in *slg-D*. Sixthly, feedback regulation on expression of the known BR genes was seen in *slg-D*. Lastly, knockdown of *SLG* resembled mild BR-deficient mutants.

The size of an organ is determined by cell proliferation and cell expansion (Potter and Xu, 2001; Sugimoto-Shirasu and Roberts, 2003). Our results showed that *SLG* is required for cell expansion in grains. To investigate the possible regulatory relationship between *SLG* and other previously identified genes that control grain size by influencing cell expansion, such as *PGL1*, *GL7*, and *GS2/GL2* (Heang and Sassa, 2012; Che *et al.*, 2015; Duan *et al.*, 2015; Hu *et al.*, 2015; Wang *et al.*, 2015), we examined the transcript level of these genes and found no obvious difference between *slg-D* and the WT (see Supplementary Fig. S12). This result suggests that *SLG* may regulate grain size in a pathway independent of *PGL1*, *GL7*, and *GS2/GL2*.

*SLG* is predicted to encode a BAHD acyltransferase-like protein. Previous studies of BAHD acyltransferase family members have shown that this family is capable of using CoA thioesters and catalyzing the formation of a wide variety of plant metabolites by generating ester or amide bonds (D’Auria, 2006). In Arabidopsis, two BAHD acyltransferases, BIA1 and BAT1, are involved in BR homeostasis, probably by conversion of active BR intermediates into inactive acylated BR conjugates (Roh *et al.*, 2012; Choi *et al.*, 2013). Overexpression of *BIA1* or *BAT1* results in decreased levels of active BRs and typical BR-deficient phenotypes (Roh *et al.*, 2012; Choi *et al.*, 2013). In our study, SLG, as a BAHD acyltransferase, probably converts an as yet unidentified substrate to the corresponding acyl conjugate to affect endogenous BR levels in an opposite way. Overexpression of *SLG* induced increased levels of active BRs and BR-overproduction phenotypes. The difference in Arabidopsis and rice implies that the function of BAHD acyltransferases in BR homeostasis has been differentiated. On the other hand, most of the enzymes known to catalyze BR biosynthesis belong to the cytochrome P450 protein family (Choe, 2006), implying that SLG, as a BAHD acyltransferase, may not work directly on the known BR intermediates, or that it may represent a different class of enzymes mediating BR synthesis. Further studies are needed to clarify how SLG participates in BR homeostasis.

In many cases, homomer formation is an essential biochemical process as it forms the complex quaternary structures of proteins to regulate selectivity against different substrates, enzyme activity, or stability (Dayhoff *et al.*, 2010). Here, we showed that SLG interacted with itself, and its N-terminal 30 amino acid region was required for the interaction. It is likely that the self-interaction of SLG forms a functional enzyme complex with a special quaternary structure that binds the target substrates effectively. A truncated protein SLG∆C1 lacking a 255 amino acid C-terminus is still able to interact with the intact version but may form a complex unable to function properly due to a change in the quaternary structure, thus leading to a dominant negative phenotype. This finding also suggests that the substrate recognition and/or catalyzing domain may be located in the C-terminus. The version without the N-terminal interaction region failed to create a dominant negative phenotype, further confirming that the homomer formation of SLG indeed exists *in vivo*. It will be interesting to investigate further the number of SLG proteins required to form a functional enzyme complex and its structural organization.

The plant architecture determines planting density, and thus yield. In rice, BR-deﬁcient or -insensitive mutants show the erect leaf phenotype, such as *d2*, *d11*, and *d61* (Yamamuro *et al.*, 2000; Hong *et al.*, 2003; Tanabe *et al.*, 2005). More erect leaves that increase light capture and thus enhance photosynthetic efﬁciency and nitrogen storage for grain filling can be combined with high planting densities to improve grain yield and biomass in rice (Sinclair and Sheehy, 1999; Morinaka *et al.*, 2006; Sakamoto *et al.*, 2006). For example, modulating the expression levels of *OsDWARF4* and *OsBRI1* led to the erect leaf phenotype and efficiently improves rice grain yield and biomass in dense planting conditions (Morinaka *et al.*, 2006; Sakamoto *et al.*, 2006). *SLG*, when knocked-down by RNAi or interfered with by a truncated version, can create a compact semi-dwarf plant type with smaller leaf angles. Therefore, *SLG* can be used as an alternative to manipulate plant height for lodging resistance and leaf angle for planting density by optimizing its expression level, offering the potential for improving rice production.

## Supplementary data

Supplementary data are available at *JXB* online.

Figure S1. The *slg-D* mutation behaves in a semi-dominant manner.

Figure S2. Co-segregation analysis of phenotypes and genotypes in F_2_ progeny.

Figure S3. Overexpression of *Loc_Os08g44830* does not phenocopy the phenotypes of *slg-D*.

Figure S4. Phylogenetic tree of SLG homologs.

Figure S5. Alignment of the monocot group of SLG homologs.

Figure S6. Sensitivities of roots and coleoptiles to BL are not altered in *slg-D*.

Figure S7. Responses of WT and *m107* leaf lamina joint angles to BRZ.

Figure S8. Measurements of endogenous BR intermediates.

Figure S9. Quantitative RT-PCR analysis of BR-related genes in young *m107* and WT panicles.

Figure S10. Quantitative RT-PCR analysis of *SLG* expression in WT seedlings treated with BL.

Figure S11. Overexpression of *SLG∆N3* does not change the phenotypes of the WT and *slg-D*.

Figure S12. Quantitative RT-PCR analysis of several genes that control grain size by influencing cell expansion in *slg-D* and the WT.

Table S1. Primers used in this study.

Supplementary Data
