# Supplementary material for: SLG controls grain size and leaf angle by modulating brassinosteroid homeostasis in rice
Source: J Exp Bot. 2016 Jun 1;67(14):4241–53. doi: 10.1093/jxb/erw204 (PMC5301929; doi:10.1093/jxb/erw204)
Supplement: Supplementary Data [file supp_67_14_4241__index.html]

 SLG controls grain size and leaf angle by modulating brassinosteroid homeostasis in rice — SLG controls grain size and leaf angle by modulating brassinosteroid homeostasis in rice — Supplementary Data 

# *SLG* controls grain size and leaf angle by modulating brassinosteroid homeostasis in rice

## Supplementary Data

Data files

- supplementary\_figures\_S1\_S12\_table\_S1.pdf - Supplementary Data
