# Supplementary material for: SLG controls grain size and leaf angle by modulating brassinosteroid homeostasis in rice
Source: J Exp Bot. 2016 Jun 1;67(14):4241–53. doi: 10.1093/jxb/erw204 (PMC5301929; doi:10.1093/jxb/erw204)
Supplement: Supplementary Data [file supp_erw204_supplementary_figures_S1_S12_table_S1.pdf]

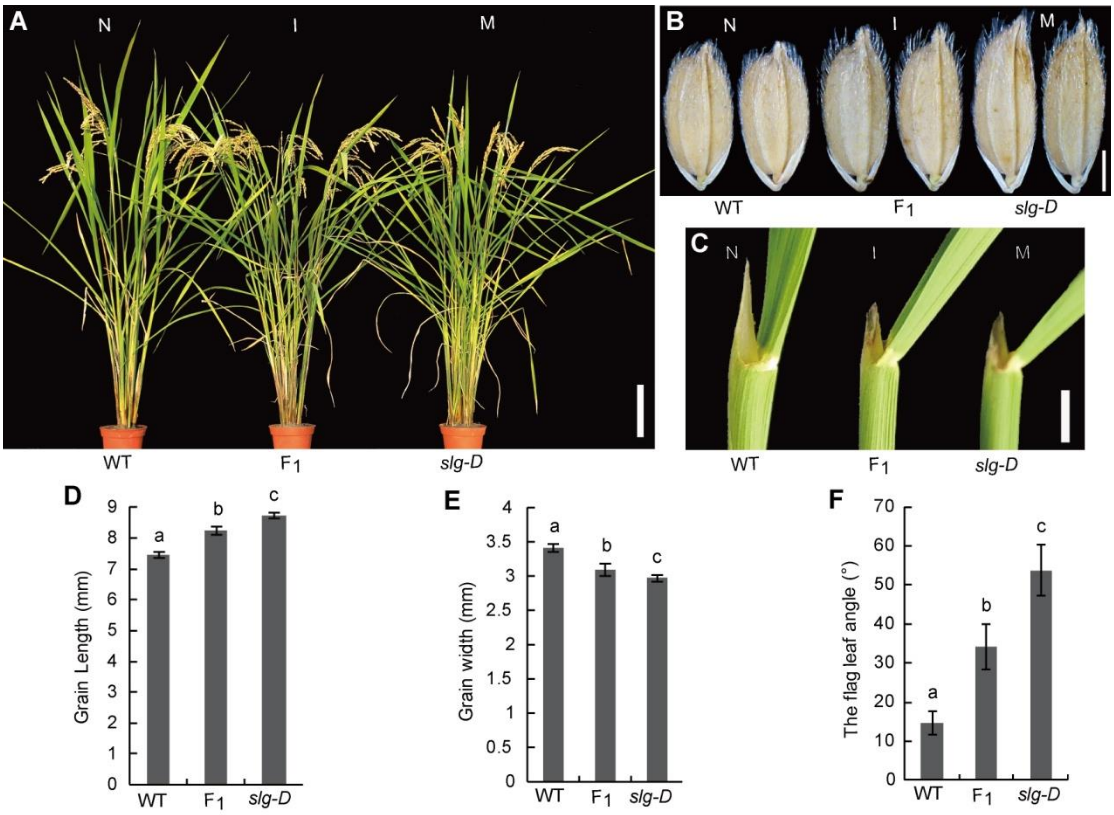

G

Population	Total	N	I	M	χ^2 (1:2:1)
slg-D / WT F_2	240	64	120	56	0.09 (*P*>0.05)

Supplementary Figure S1. The *slg-D* mutation behaves in a semi-dominant manner. (A-F) Comparison of WT, heterozygous (F_1) and homozygous *slg-D* plants at the maturation stage (A), grains (B), the lamina joints of the flag leaves (C), grain lengths (D), grain widths (E) and the lamina joint angles of the flag leaves (F). Values are given as means ± SD (n=10 in D-F). Different letters indicate $P< 0.05$ (LSD multiple range tests). Scale bars = 10 cm (A), 2 mm (B) or 2 cm (C). N, I and M indicate normal, intermediate and mutant phenotype, respectively. **(G)** Segregation of F_2 progeny from a self-pollinated F_1 plant (*slg-D* × WT).

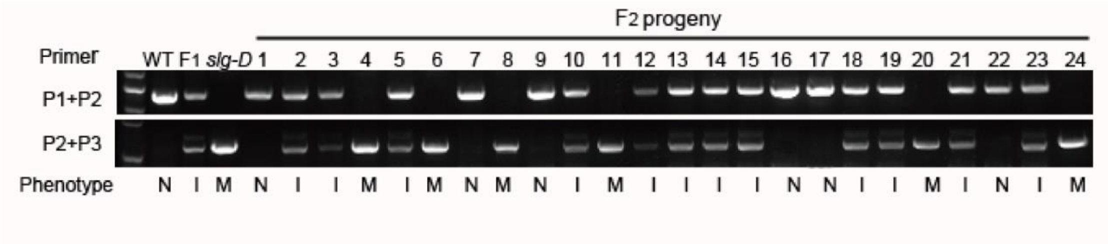

Supplementary Figure S2. Co-segregation analysis of phenotypes and genotypes in F_2 progeny. 1-24 are twenty-four F_2 plants generated by crossing *slg-D* with WT. N, I and M indicate normal, intermediate and mutant phenotypes, respectively.

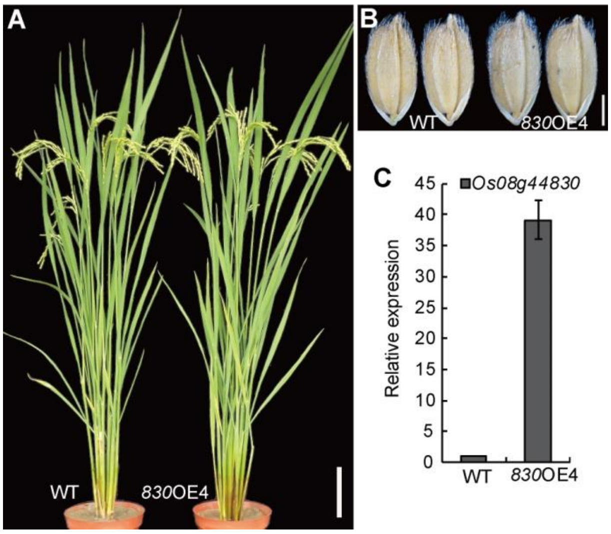

Supplementary Figure S3. Overexpression of *Loc_Os08g44830* doesn't phenocopy the phenotypes of *slg-D*. (A) Gross phenotype of *pUbi::Loc_Os08g44830* transgenic plants (*830*OE4) in the WT background. **(B)** Grains of plants shown in (A). **(C)** Expression level of *Loc_Os08g44830* in the plants shown in (A). Values are given as means ± SD (n=3). Scale bars = 10 cm (A) or 2 mm (B).

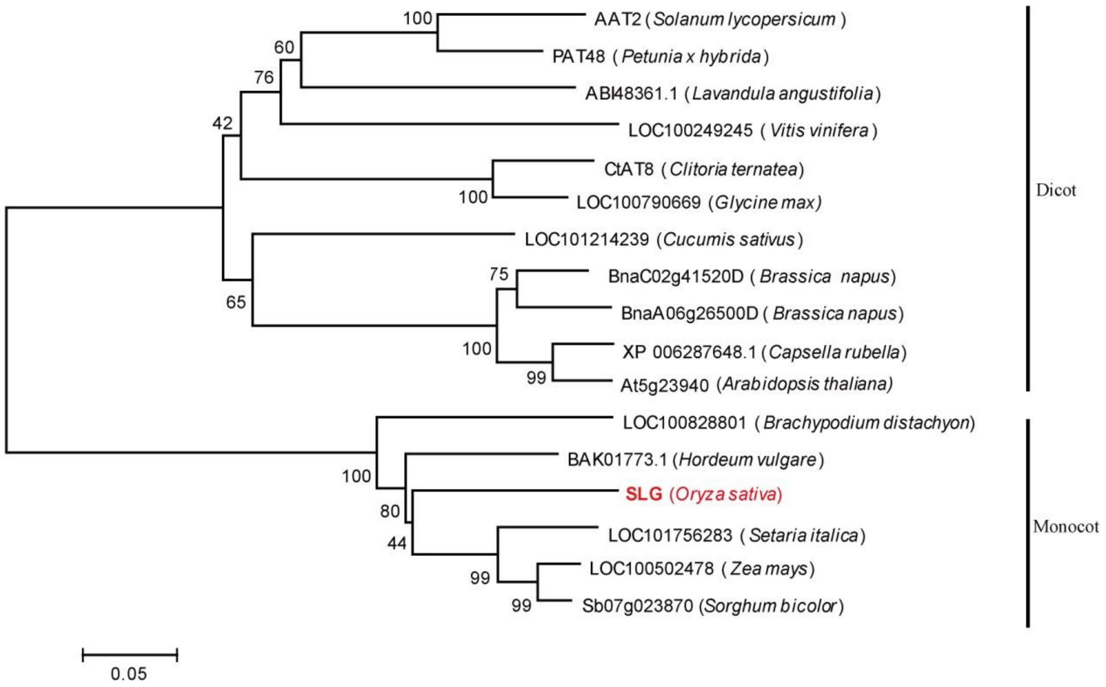

Supplementary Figure S4. Phylogenetic tree of SLG homologs. Phylogenetic tree derived from protein sequence was constructed using the neighbor-joining method of the MEGA4.0 program. Bootstrap values from 1,000 replicates are indicated at each node. The 0.05 scale shows substitution distance.

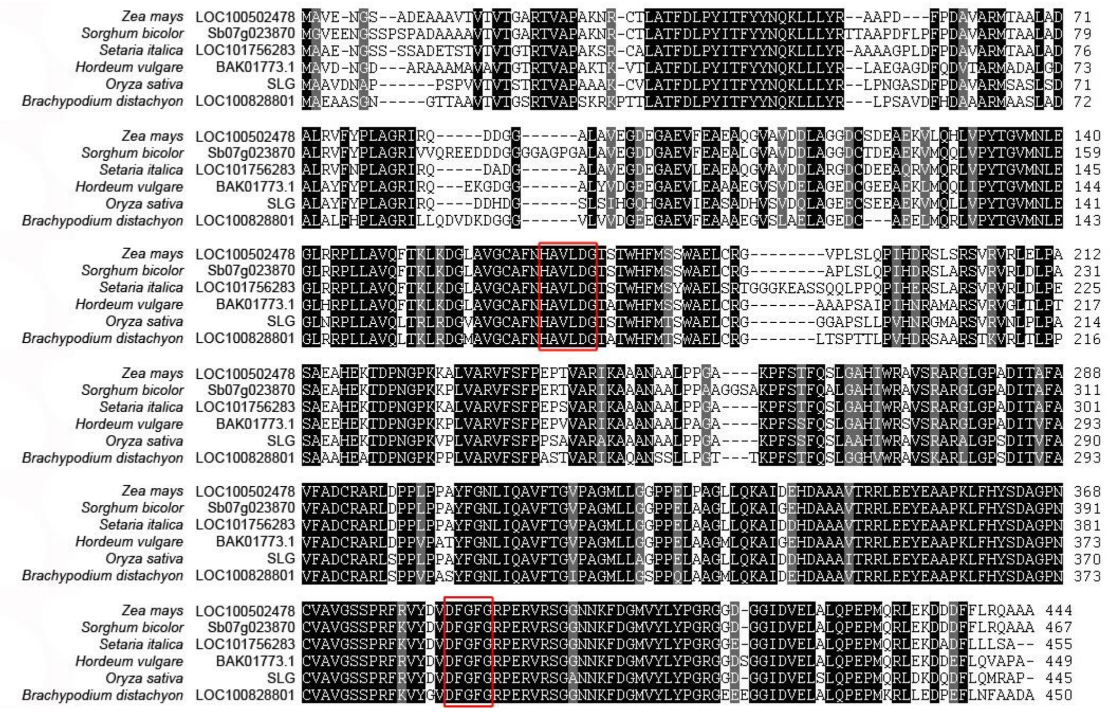

Supplementary Figure S5. Alignment of the monocot group of SLG homologs. The multiple sequence alignment was performed using the CLUSTALW analysis tool. Amino acid residues identical and similar to those of SLG are shaded in black and gray, respectively. The red boxes indicate the two conserved motifs.

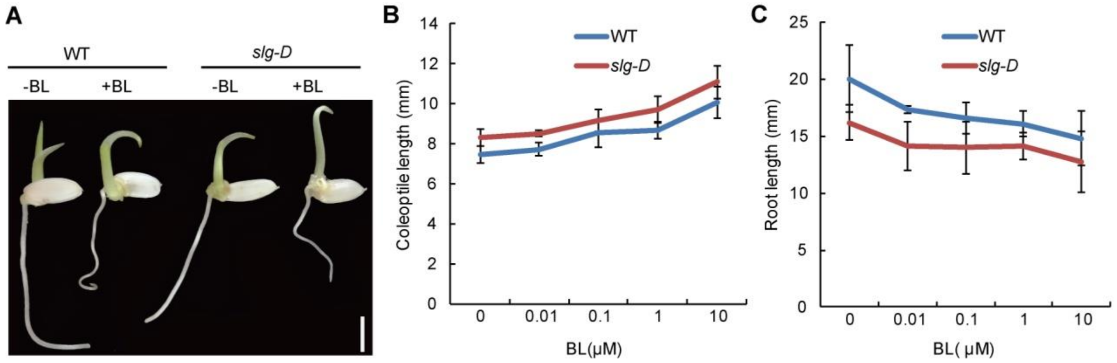

Supplementary Figure S6. Sensitivities of roots and coleoptiles to BL are not altered in *slg-D*. (A) Seeds were germinated on agar medium in the presence (-) or absence (+) of 10 μM BL. Seedlings were examined 1 day after germination. Scale bar = 2 mm. **(B, C)** Effects of BL on the coleoptile (B) and root (C) elongation in seedlings. The plants were germinated in the same conditions as (A) with the indicated concentrations of BL. Values are given as means ± SD (n=5 in B, C).

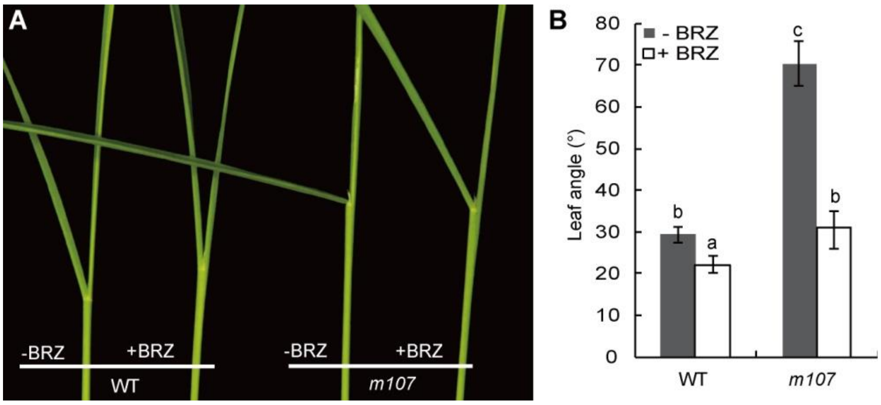

Supplementary Figure S7. Responses of WT and *m107* leaf lamina joint angles to BRZ. (A) Response of the third leaf lamina joint from WT and *m107* plants to 10 μM BRZ. **(B)** Measurement of WT and *m107* lamina joint inclinations after the 10 μM BRZ treatment shown in (A). Values are given as means ± SD (n=10). Different letters indicate *P*< 0.01 (LSD multiple range tests).

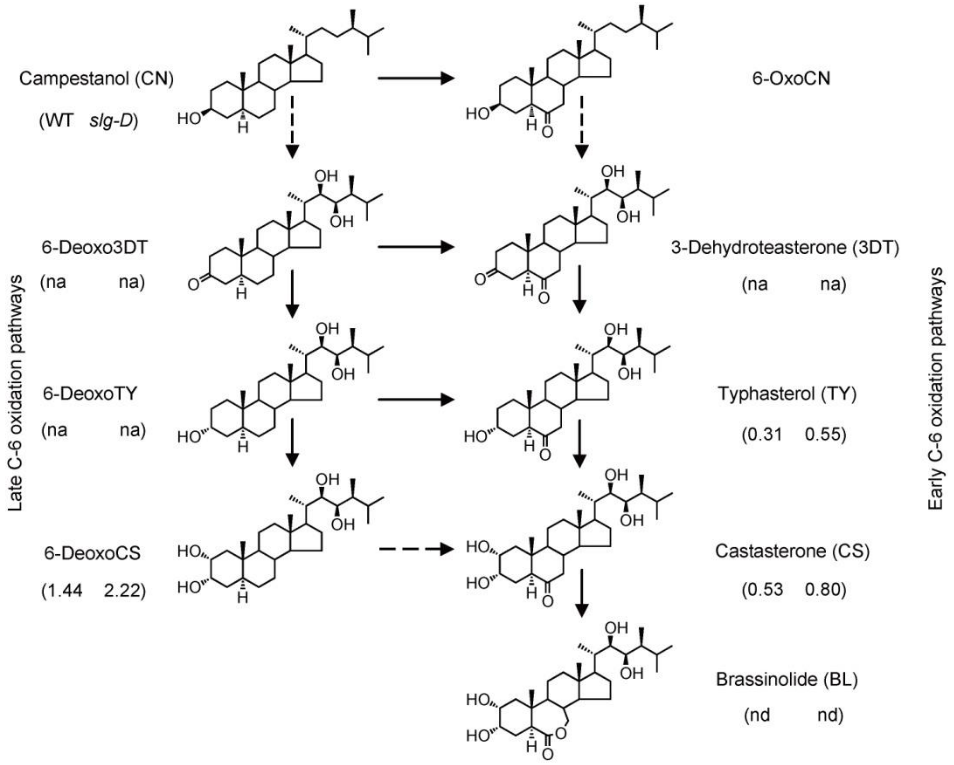

Supplementary Figure S8. Measurements of endogenous BR intermediates. BR levels (ng/g fresh weight) in shoots of WT (left) and *slg-D* (right) plants are shown below each product. na, not analyzed; nd, not detected.

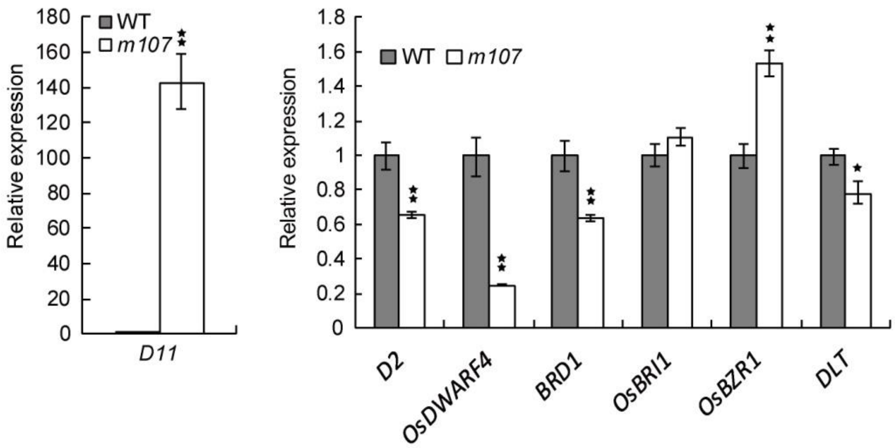

Supplementary Figure S9. Quantitative RT-PCR analysis of BR-related genes in young *m107* and WT panicles. Values are given as means ± SD (n=3). *P < 0.05; **P < 0.01 compared with WT by Student's t-test.

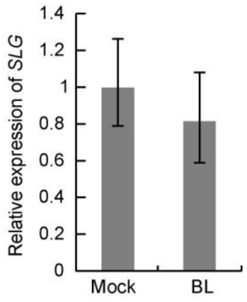

Supplementary Figure S10. Quantitative RT-PCR analysis of *SLG* expression in WT seedlings treated with BL. 7-days-old rice seedlings were soaked in water containing no hormone (Mock) or 10 μM BL for 12h. Values are given as means ± SD (n=3).

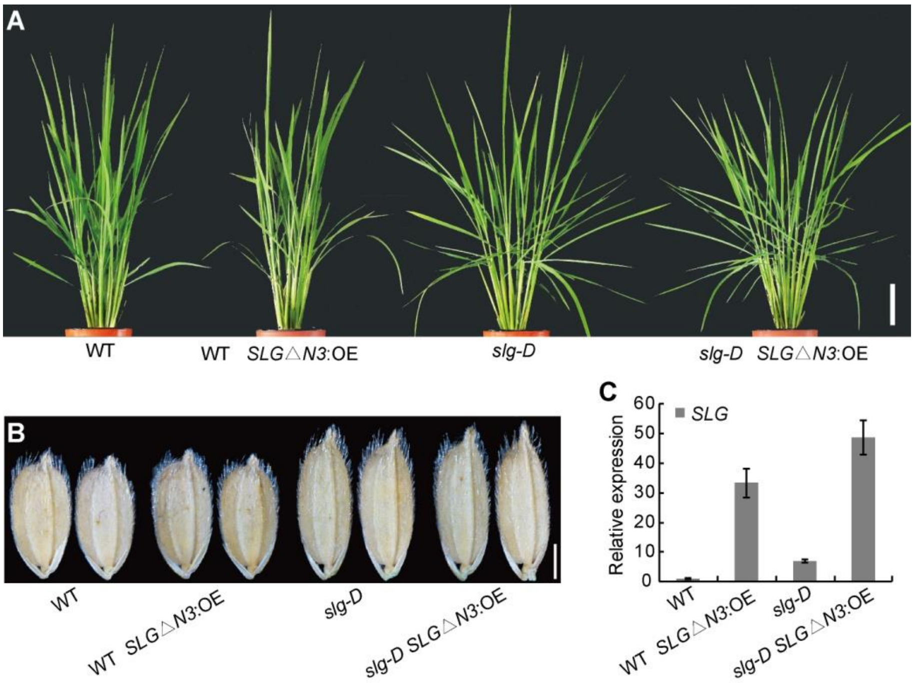

Supplementary Figure S11. Overexpression of *SLG∆N3* doesn't change the phenotypes of WT and *slg-D*. (A, B) Gross morphologies (A) and grains (B) of WT, WT *SLG∆N3*:OE, *slg-D* and *slg-D SLG∆N3*:OE. (**C)** Expression levels of *SLG* in the lines shown in (A). Values are given as means ± SD (n=3). Scale bars = 10 cm (A) or 2 mm (B).

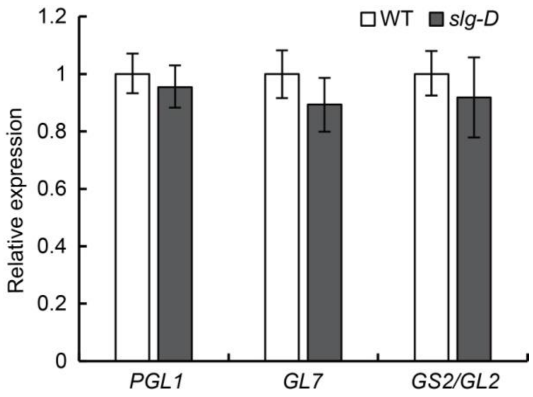

Supplementary Figure S12. Quantitative RT-PCR analysis of several genes that control grain size by influencing cell expansion in *slg-D* and WT. Values are given as means ± SD (n=3).

Supplementary Table S1. Primers used in this study.

Purpose	Primer name	Primer sequence(5'-3')
Genotyping	P1	GGCACTGACCTCAATAGTTTCG
	P2	TTCCATTACCACCTTCATCCC
	P3	AAGTCCCGCTAGTGCCTTGT
Quantitative	q840/SLG F	CAAGTTCGACGGGATGGTCTACCT
RT-PCR	q840/SLG R	CTCGCATTTGGAGGAAGTCTTGG
	q830 F	CAGCCGCCAGCACGATTACA
	q830 R	CTTGAACCCCTTGCCGCACA
	q820 F	TGACGAGGATGTTGGTTTGC
	q820 R	GAATGGTTGCTGTTGTTTGA
	qActin F	TCCATCTTGGCATCTCTCAG
	qActin R	GTACCCGCATCAGGCATCTG
	qD2 F	CCTTTTGGTGGTGGGCAGAG
	qD2 R	TGGGGAAGTTGACGATGTGGT
	qD11 F	CAAGGGACAAGCAAGAAGTTTAC
	qD11 R	CGATTTCTATGGGCAGACCTC
	qOsDWARF4 F	TGGGCTCTGAAACAATCTAACCT
	qOsDWARF4 R	CAAGGAAGAAGATGGCGAGG
	qBRD1 F	GAGGTGGCTGGAGAAGAACAT
	qBRD1 R	ATTTCTACGGTGCCTACTTCCT
	qOsBRI1 F	GCAAGGGTATCTGATTTCGGT
	qOsBRI1 R	CAAGAGTGGACACGCTAAGGT
	qOsBZR1 F	AGATGGTTCCTTTCGTGGAC
	qOsBZR1 R	AGAATGAAATCGCCCAAATC
	qDLT F	TGCGGATACTCAACGCCATCA
	qDLT R	ACTCGCCGACTCCGGTGATC
	qPGL1 F	ATGTCAAGCCGGAGGTCACG
	qPGL1 R	CGACTCCGGAGCAATGATAGA

	qGL7 F	GGGACACCGGAGGCCTTA
	qGL7 R	TGCCCATTCCTCCTTGCAT
	qGS2/GL2 F	AGACCTCGCTGATGAGAATG
	qGS2/GL2 R	AGCAACAAGGCCAGTATGAG
Overexpression	830-1390 F	TTCTGCACTAGGTACCATGATTCCAGGCGGTGGCGG
	830-1390 R	GGACTAGTTCAGGCGGCGCCCATGCCAA
	840-1390 F	TTCTGCACTAGGTACCATGGCGGCCGTCGACAATGC
	840-1390 R	GGACTAGTTTAAGGGGCTCGCATTTGGA
	SLGΔC1-2300 F	GTAGAAGAGGTACCCGGGATGGCGGCCGTCGACAATGC
	SLGΔC1-2300 R	GCCCTGGCATGCCTGCAGTTAGCCGCCGCCCCGGCAGAG CT
	SLGΔN3-2300 F	GTAGAAGAGGTACCCGGGGCCACATTCGACCTCCCCTA
	SLGΔN3-2300 R	GCCCTGGCATGCCTGCAGTTATTAAGGGGCTCGCATTTGGA
RNAi	SLG-RNAiL F	TTCTGCACTAGGTACCACCATGTCTCCGTCGACCAA
	SLG-RNAiL R	CTGACGTAGGGGCGATAGAGCTCAGGGGCACCTTGGGCC CATT
	SLG-RNAiR F	CGGGGATCCGTCGACTACACCATGTCTCCGTCGACCAA
	SLG-RNAiR R	AGGTGGAAGACGCGTTACAGGGGCACCTTGGGCCCATT
GUS staining	SLG-GUS F	CCGGAATTCAGAAACCCTTAGATTAGATGCC
	SLG-GUS R	ACGCGTCGACGCTGATGGTATGGTAGGGGA
In situ hybridization	SLG-P F	ACCATGTCTCCGTCGACCAA
	SLG-P R	GTTGACGCGGACGGAGCGC
Subcellular localization	SLG-GFP F	GGACTAGTATGGCGGCCGTCGACAATGC
	SLG-GFP R	CGCGGATCCAGGGGCTCGCATTTGGA
Yeast two-hybrid assay	SLG-BD F	GGAATTCCATATGATGGCGGCCGTCGACAATGC
	SLG-BD R	CCGGAATTCTTAAGGGGCTCGCATTTGGA
	SLG-AD F	GGAGGCCAGTGAATTCATGGCGGCCGTCGACAATGC
	SLG-AD R	CACCCGGGTGGAATTCTTAAGGGGCTCGCATTTGGA
	SLGΔC1-AD R	CACCCGGGTGGAATTCGCCGCCGCCCCGGCAGAGCT

	SLGΔC2-AD R	CACCCGGGTGGAATTCGGGGACGAGGAGCTGCATCA
	SLGΔC3-AD R	CACCCGGGTGGAATTCGGAGGCGCCGTTGGGGAG
	SLGΔC4-AD R	CACCCGGGTGGAATTCGAGGACGCACTTGGCGGC
	SLGΔN1-AD F	GGAGGCCAGTGAATTCGCGCCGTCGCTGCTGCCG
	SLGΔN2-AD F	GGAGGCCAGTGAATTCCTCCTCGTCCCCTACACC
	SLGΔN3-AD F	GGAGGCCAGTGAATTCGCCACATTCGACCTCCCCTA
Pull-down assay	MBP-SLG F	AGGATTTCAGAATTCATGGCGGCCGTCGACAAT
	MBP-SLG R	GCCAGTGCCAAGCTTTTAAGGGGCTCGCATTTGG
	GST-SLG F	CGCGTGGATCCCCGGAAATGGCGGCCGTCGACAATGC
	GST-SLG R	GTCGACCCGGGAATTCTTAAGGGGCTCGCATTTGGA
BiFC assay	cYFP-SLG F	CGCCACTAGTGGATCCATGGCGGCCGTCGACAATG
	cYFP-SLG R	TACTATCGATGGATCCAGGGGCTCGCATTTGGAGGA
	nYFP-SLG F	CGCCACTAGTGGATCCATGGCGGCCGTCGACAATG
	nYFP-SLG R	TACTATCGATGGATCCAGGGGCTCGCATTTGGAGGA
	nYFP-SLGΔC4 R	TACTATCGATGGATCCGAGGACGCACTTGGCGGC
	nYFP-SLGΔN3 F	CGCCACTAGTGGATCCGCCACATTCGACCTCCCCTA
